# Growth of ZnO Nanorods on ITO Film for Piezoelectric Nanogenerators

**DOI:** 10.3390/ma14061461

**Published:** 2021-03-17

**Authors:** Hyun Gi Kim, Eun Hye Kim, Sung Soo Kim

**Affiliations:** 1 Department of Chemical Engineering, Kyung Hee University, Yongin 17014, Korea; opti_people@khu.ac.kr; 2 Core Facility Center for Analysis of Optoelectronic Materials and Devices, Kyung Hee University, Yongin 17104, Korea; skysm@khu.ac.kr

**Keywords:** zinc oxide nanorod, ZnO seed layer, piezoelectric, nanogenerator, energy harvesting

## Abstract

Piezoelectric nanogenerators (NGs) consist of zinc oxide nanorods (ZNRs), and polydimethylsiloxane (PDMS) layers were fabricated on indium tin oxide (ITO)-coated substrate for the energy harvesting system. The formation of seed layers by an optimized aqueous solution method greatly helped the growth of well-aligned ZNRs for NGs. Polyethylenimine (PEI) was added to increase the aspect ratio of ZNRs, which reached up to 24:1, showing the best energy harvesting performance of NGs. The formation of PDMS layers on the ZNRs increased the work function difference for the top Ag electrode. The thickness of PDMS layers was optimized as 80 μm, which showed the maximum work function difference, resulting in the enhancement of charge density. Piezoelectric NGs made of ZNRs of the highest aspect ratio of 24:1 with an 80-μm-thick PDMS layer achieved the highest current density of 2270.1 nA/cm^2^, which could be sufficient to drive low-power electronics.

## 1. Introduction

Piezoelectric nanogenerators (NGs) have been developed for energy harvesting systems [1]. By utilizing the piezoelectric properties of ferroelectric materials, they can harvest the electrical energy from the mechanical energy of natural sources such as wind, waves, or vibration [2]. Several materials with nanostructures have been extensively investigated for utilization in self-powered energy harvester and piezoelectric devices [3,4]. A single crystal of zinc oxide (ZnO) is known to have significantly fast electron transport properties as well as great mobility. The fast electron transport comes from the high electron diffusion coefficients, which provides significant advantages in terms of device performance [5]. Since ZnO has a large surface to volume ratio, which leads to the enhancement of the sensitivity, it is an excellent material for sensor application. ZnO is one of the piezoelectric materials which can transform the mechanical stress/strain into electrical voltage due to the relative displacement of the cations and anions in the crystal [6].

ZnO can be prepared in various forms, such as nanoparticles [7], nanorods [8], and nanowires [9], and it has gained great attention due to its important characteristics, including piezoelectricity, transparency, biocompatibility, and ease of fabrication at low temperature and with large area. Since ZnO nanowires (ZNWs) were first used for NG applications in 2006, many research groups have demonstrated the piezoelectric properties of NGs using ZnO [10,11]. Lu et al. fabricated piezoelectric NGs using p-type ZNW arrays on a silicon substrate [9]. They reported energy conversion using p-type ZNWs for the first time. Liu et al. grew well-aligned ZNWs on a GaN/AlN substrate through a vapor–liquid process and analyzed the factors of the nanowires that determined the power output of a piezoelectric NG [12]. Greene et al. fabricated dense arrays of ZNWs with high surface area on arbitrary substrates of variable size and concluded that the use of homogeneous ZnO nanocrystals as a seed layer helps to produce dense ZNWs on the substrate [13].

It is difficult to ensure that ZNWs are uniformly ordered during device fabrication and a more ordered structure is required for the performance enhancement. Nanorods can be used in a more ordered structure formation than the other forms of ZnO. ZnO nanorods (ZNRs) have large surface areas to be characterized for gas and chemical sensing as well as piezoelectric applications. A highly oriented array of ZNRs can be produced via various deposition techniques, such as chemical vapor deposition (CVD) [14] or metal organic CVD (MOCVD) [15], vapor–liquid–solid (VLS) growth [16], electrochemical deposition (ED) [17], and hydrothermal approaches. The hydrothermal method [18,19] has attracted considerable attention due to its unique advantages. It is a simple and low-temperature process with high yield at low cost. It is an uncomplicated, controllable process to produce well-defined structures with excellent morphology.

Zn salt and hexamethylenetetramine make a chemical precursor solution for ZNR formation via the hydrothermal route, which is prepared on Si substrates with a seed layer prepared from zinc acetate solution. The effects of the seed layer on ZNR growth using the hydrothermal method have been investigated by several research groups [20,21]. ZnO seed layers with high c-axis orientations and smoother surfaces lead to well-aligned ZNR arrays with uniform diameters and narrow distributions [21]. It was reported that the formation of a seed layer enhanced the ZNR growth for its application to solar cells [22], and its application to NGs should also be examined.

The effect of polyethyleneimine (PEI) on the structure and micro-morphology of ZnO nanorod array films, as well as the photoelectric conversion properties in dye-sensitized solar cells (DSSCs), was analyzed. It was found that with the addition of PEI into the growth solution, the ZNRs became smaller in diameter and longer in length; hence, the dye absorption and the photovoltaic performance of DSSCs were improved. The effects of PEI on ZnO nanorods for piezoelectric applications have not been investigated and will be examined in this work in terms of the aspect ratio of formed ZnO nanorods [6].

Thakura et al. synthesized a highly dielectric ZnO/PVDF composite thin film via an in situ process for energy harvesting and energy storing applications. It can be used for self-poled piezoelectric nanogenerators and self-charged photo-power banks with high durability [7]. They achieved superiority in terms of power generation and energy storage capability compared to other similar prototype devices reported elsewhere. However, in the ZnO/PVDF composite thin film, it was difficult to achieve well-ordered ZnO structures.

In this work, we fabricated a dense ZNR array on substrates using a hydrothermal method. To control the aspect ratio of ZNRs, PEI was introduced into the precursor solution. A PMDS layer was formed above the ZnO nanorod layer with proper thickness, instead of impregnating the ZnO nanoparticles in a polymeric matrix. Piezoelectric NGs were fabricated using ZNRs with various aspect ratios to examine the effect of the aspect ratio on the performance of the NGs. We also attempted to demonstrate that PDMS layer formation enhances the current density of the device in terms of the maximum work function difference for the top electrode.

## 2. Materials and Methods

### 2.1. Materials

Zinc acetate dihydrate (Zn (CH_3_COO)_2_·2H_2_O, 98%, Sigma-Aldrich, St. Louis, MO, USA) and ethyl alcohol (CH_3_CH_2_OH, 99.5%, Sigma-Aldrich, Youngin, Korea) were used for preparation of the seed layer. For growth of ZNRs, zinc nitrate hexahydrate (Zn (NO_3_)_2_·6H_2_O, 98%, Sigma-Aldrich, Youngin, Korea) and hexamethylenetetramine (C_6_H_12_N_4_, 99%, Sigma-Aldrich, Youngin, Korea) were used. In addition, PEI (H(NHCH_2_CH_2_)_n_ NH_2_, average M_w_ ~800, Sigma-Aldrich, Youngin, Korea) was used as a surfactant to increase the aspect ratio of ZNRs. PDMS (Sylgard 184, premixed with curing agent in a ratio of 10:1 *w*/*w* and degassed) was purchased from Dow Corning Co. Polyethersulfone (PES) film deposited with indium tin oxide was used as substrate for fabrication of NGs.

### 2.2. Sample Preparation

#### 2.2.1. Preparation of the ZnO Seed Layer

ZnO seed layers were fabricated using an aqueous solution method. For preparation of the seed layer solution, 25 mmol of zinc acetate dihydrate was dissolved in 50 mL of ethanol as precursor. This solution was stirred for 30 min in an ultrasonic bath at room temperature to obtain an agglomerate-free solution. Additionally, the solution was heated to 90 °C and stirred for 10 min to fully dissolve the precursor. The indium tin oxide-coated polyethersulfone (ITO/PES) substrate was cleaned in an isopropyl alcohol ultrasonication chamber and thoroughly dried at 80 °C in an oven, and it was treated in argon plasma for 3 min for contaminant removal and wettability enhancement. The seed solution was spin-coated on the substrate at a speed of 1000 rpm for 60 s. Then, the substrates were post-annealed at 100 °C for 2 min on a hot plate for removal of residual solvent and organics by evaporation. To optimize the density of the seed layer, this coating process was repeated several times.

#### 2.2.2. Growth of ZNRs

ZNRs were grown in aqueous solution using a hydrothermal method. First, 25 mmol of zinc nitrate hexahydrate and 25 mmol of hexamethylenetetramine were dissolved in 300 mL of deionized water and stirred at 75 °C to prepare the aqueous solution. ITO/PES substrate coated with seed layer was immersed in aqueous solution for 3, 6, or 9 h and heated in a hot air oven at a constant temperature of 90 °C for ZNR growth according to the series of reactions presented below [23]:

Decomposition reaction: (CH_2_)_6_N_4_ + 6H_2_O → 6HCHO + 4NH_3_

Hydroxyl supply reaction: NH_3_ +H_2_O ↔ NH^4+^ +OH^-^

Supersaturation reaction: 2OH^-^ +Zn^2+^ → Zn(OH)_2_

ZnO nanorod growth reaction: Zn(OH)_2_ → ZnO + H_2_O

Next, 1.5 mmol of PEI was added to the prepared precursor solution to examine its effects on the aspect ratios of ZNRs. Then, the grown ZNR samples were rinsed in a deionized water ultrasonic chamber and fully dried in an oven at 100 °C.

### 2.3. Characterization

The seed layer formation on ITO/PES substrates and the growth of ZNRs were confirmed by using high-resolution field emission scanning electron microscopy (HR FE-SEM, Carl Zeiss, Merlin, Germany) and atomic force microscopy (AFM, Veeco, Dimension 3100, Santa Barara, CA, USA). X-ray diffraction (XRD) patterns were obtained by using an X-ray diffractometer (D8 Advance, Bruker, Billerica, MA, USA) equipped with a Cu Kα tube and Ni filter (k = 0.1542 nm). The work function was measured by using a scanning Kelvin probe microscope (SKP5050, KP Tech. Ltd., Caithness, Scotland). Most of the apparatus and equipment was supported by the Core Facility Center for Analysis of Optoelectronic Materials and Devices of the Korea Basic Science Institute (KBSI).

## 3. Results

**Figure 1:**
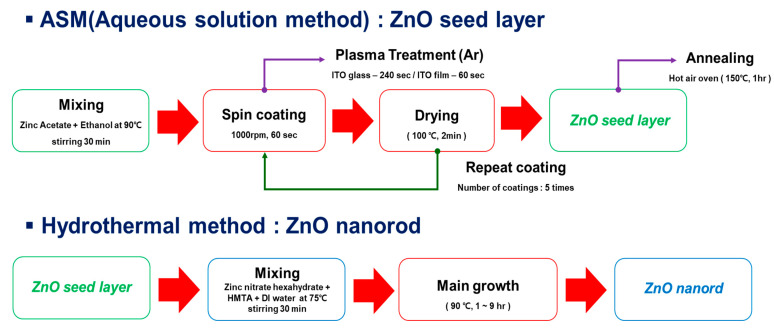
Procedures for ZnO seed layer formation by aqueous solution method and zinc oxide nanorod (ZNR) growth by hydrothermal method.

The growth of ZNRs is influenced by crystallinity, orientation, surface roughness, and ZnO seed layer. A ZnO seed layer was prepared on the ITO/PES substrate using an aqueous solution method (ASM) as illustrated in Figure 1.

**Figure 2:**
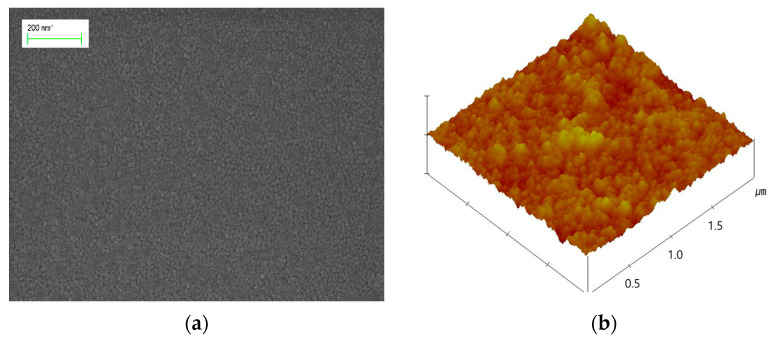
(**a**) High-resolution field emission scanning electron microscopy (HR FE-SEM) and (**b**) atomic force microscopy (AFM) images of the zinc oxide nanorod (ZnO) seed layer.

In Figure 2a, ZnO grains were found in the SEM image of the seed layer formed on the ITO/PES substrate. Even though the seed layers were formed at a relatively low temperature, annealing the samples at 150 °C enabled grain growth of uniform size on the surface of the substrate due to coalescence between the grains. The average grain size was approximately 10 nm. The ZnO seed layer led to the growth of vertically well-aligned ZNRs [21]. The AFM images of the seed layer formed on the ITO surface are shown in Figure 2b and show a smooth surface with a roughness of 1.999 nm.

ZNRs were grown uniformly on the ZnO seed layer by a hydrothermal method, and details of the procedure are shown in Figure 1. The ZnO seed layer, which plays an important role as a nucleation center for ZNRs, should have monolayer-distributed surface properties. It was expected that ZNRs would be efficiently grown on the seed layer [24].

**Figure 3:**
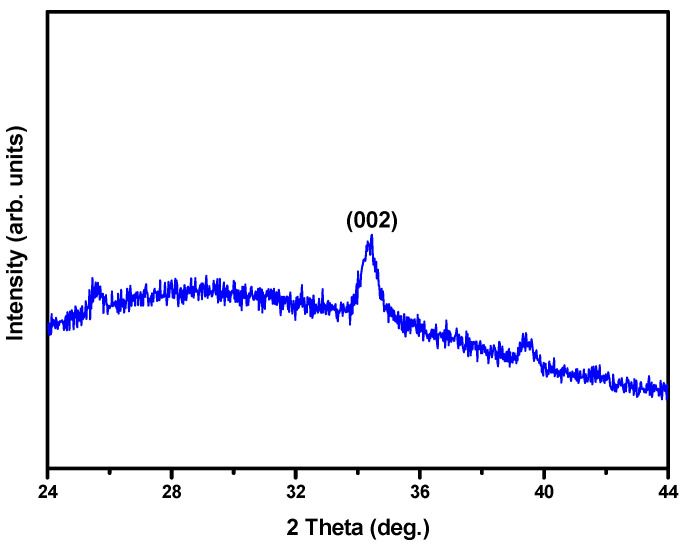
X-ray diffraction (XRD) pattern of the ZnO seed layer.

Additionally, (002) diffraction peaks were obtained for the seed layer, as shown in the XRD patterns in Figure 3. These results indicate that the seed layer has a preferred c-axis growth orientation with a polycrystalline phase.

**Figure 4:**
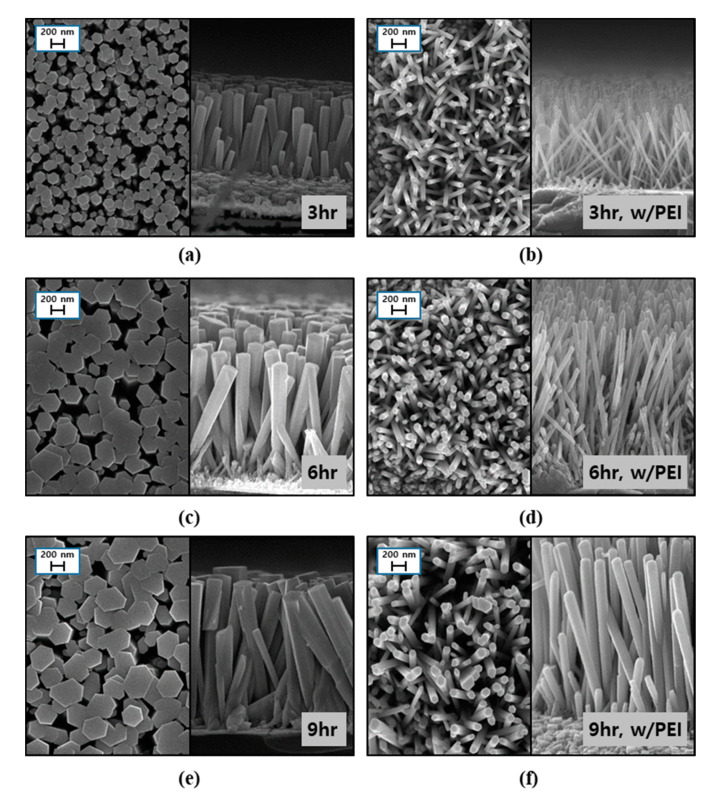
HR FE-SEM images of ZNRs prepared with various growth times at 90 °C (**a**,**c**,**e**); no polyethylenimine (PEI) and (**b**,**d**,**f**); PEI added to precursor solutions.

To investigate the effects of growth time on the dimensions of ZNRs, we fabricated ZNRs on seed layers formed on the substrate under optimum conditions. The top and cross-sectional images of the ZNRs were obtained for different growth times in the range of 3 to 9 h, as shown in Figure 4. Due to the seed layer having an orientation along the c-axis, the hexagonal shape on the top of the ZNRs could be obtained. It also could be confirmed that the ZNRs were quite uniform and vertically well-aligned, with high density on the seed layer. With an increase in growth time, both the diameter and length of ZNRs increased, as shown in Figure 4a,c,e. When PEI was added to the precursor solution, the length of ZNRs increased while the diameter decreased for every growth time, as shown in Figure 4b,d,f. Therefore, we could conclude that PEI had significant effects on the aspect ratio increase in ZNRs [24,25].

**Figure 5:**
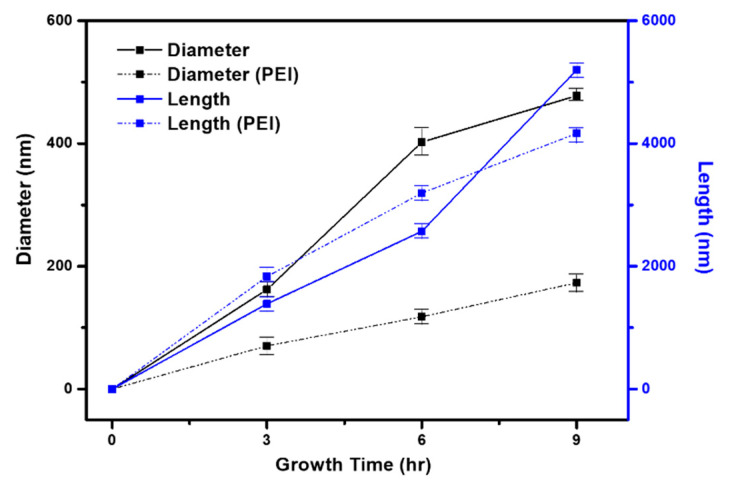
Diameter and length results of ZNRs prepared with various growth times at 90 °C for two cases: no PEI and PEI added to precursor solutions.

ZNR growth behaviors depending on the presence of PEI are compared in Figure 5. In both cases, when time in the precursor solution was increased, ZNRs had a longer diameter and length due to supplementation of ion sources from the precursors. A continuous ZnO network could not be formed on the relatively poor crystalline seed layer due to limited nuclei [16]. However, even with a lower substrate temperature of 90 °C, ZNRs were successfully grown on the seed layer of the ITO/PES substrate. These results indicate that the seed layer has relatively good crystallinity for the growth of ZNRs.

The effects of PEI addition on the growth behaviors of ZNRs over time at 90 °C were examined and the diameters and lengths of ZNRs are summarized in Table 1. The length and diameter of ZNRs increased with growth time for both cases: with and without PEI. The addition of PEI slightly increased the length of ZNRs but greatly reduced the diameters when compared to the case with no PEI. Therefore, the aspect ratio was greatly increased by the addition of PEI for every growth time result, and it reached up to 24:1.

**Figure 6:**
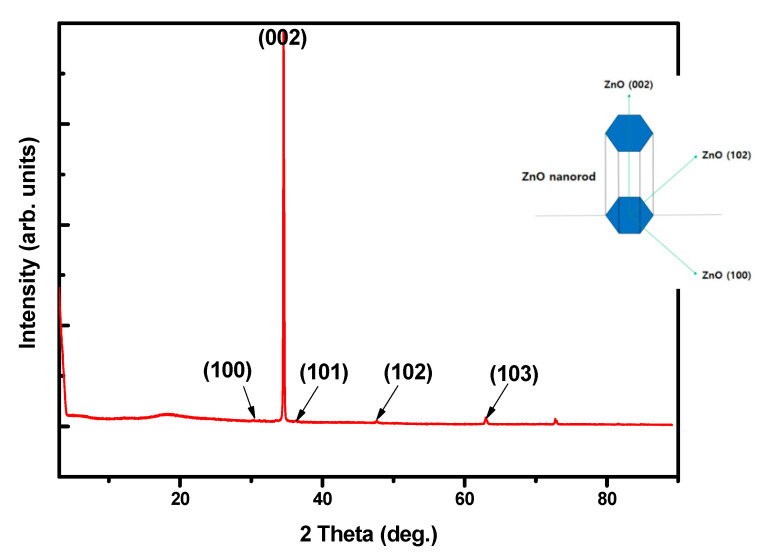
XRD pattern of the ZnO nanorod with the highest aspect ratio of 11:1.

The reason for the observed changes in PEI is that the protonated form of liner PEI inhibits the lateral growth of ZNRs. PEI contains a large number of amino groups that can be radially protonated in long molecular facts between pH values of 3 and 11. The protonated amino groups form on the nonpolar side wall of ZNRs due to electrostatic affinity. Consequently, Zn^2+^ and OH^-^ ions were continuously supplied from the precursors to the top of the ZNRs, resulting in growth along the c-axis. It was confirmed that the addition of PEI to the precursor solution was quite effective in enhancing the aspect ratio of ZNRs grown under the same conditions. Figure 6 shows the XRD of as-grown ZNRs in the presence of PEI on the ITO/PES substrate with a maximum aspect ratio of 24:1. A highly intense peak at 2θ = 34.4° corresponded to the (002) plane, demonstrating crystal growth of hexagonal nanorods oriented perpendicular to the substrate along their c-axis (as shown in the Figure 6 insert).

**Figure 7:**
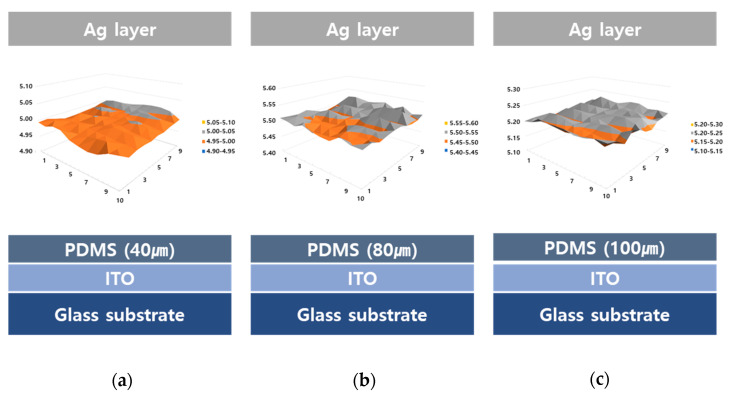
Work function distribution of polydimethylsiloxane (PDMS) layers of various thickness, (**a**) 40, (**b**) 8, (**c**) 100 μm, on indium tin oxide-coated (ITO) substrate.

The PDMS layers formed on the substrates played a role in adjusting the work function of the surface. To confirm the adjustment of the surface properties, PDMS layers with different thicknesses of 40, 80, and 100 μm were prepared on the ITO substrates without the ZNR layer. The work functions of the three samples were determined using scanning Kelvin probe microscopy. As shown in Figure 7, the level of work function was quite uniform for each thickness.

The greater difference in work function between the two different surfaces enhanced the surface charge [26]. The work function of the top Ag electrode layer was 4.64 eV. The difference in work function between the top Ag electrode and PDMS layer was determined for each PDMS thickness and these values are summarized in Table 2. The work function of the 80-μm-thickness sample was greater than that of the other samples. The work function of the 40-μm-thickness sample might have been affected by the ITO surface with 4.6 eV of work function. In the case of the PDMS layer with 100 μm thickness, it was expected that a thicker PDMS layer would minimize the degradation of ZNRs. However, the work function difference slightly decreased. Therefore, the PDMS was optimized to obtain the maximum difference in the work function. From these points of view, it was inferred that the current density of the PDMS surface increased due to the larger difference in work function for the top Ag electrode.

**Figure 8:**
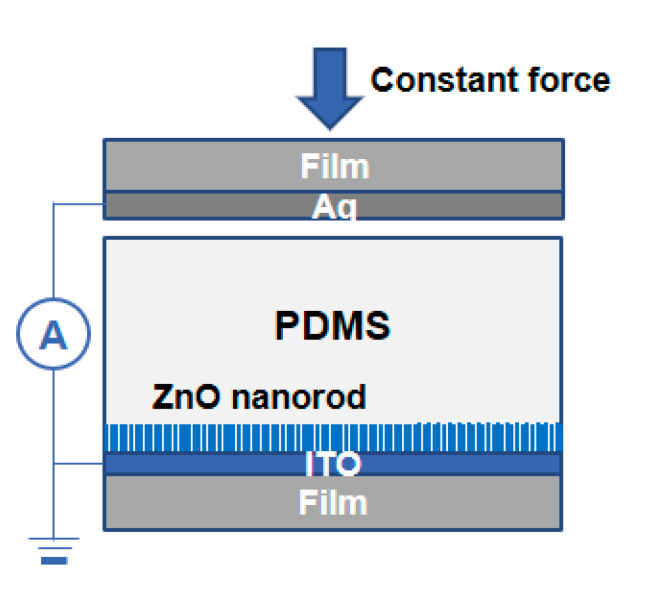
A schematic of test device fabrication of piezoelectric nanogenerators (NGs) for evaluation of power generation.

To evaluate power generation, a test device of piezoelectric NGs was fabricated, as shown in Figure 8. The current densities generated by piezoelectric NGs with a top layer of Ag/PES film were measured under a compressive force of 1 N. The size of piezoelectric NGs was 3 × 5 cm^2^; however, the active area was reduced to 0.7 × 0.7 cm^2^. To minimize degradation of ZNRs, PDMS layers of various thickness were spin-coated on top of the nanorod layer with an aspect ratio of 11:1.

**Figure 9:**
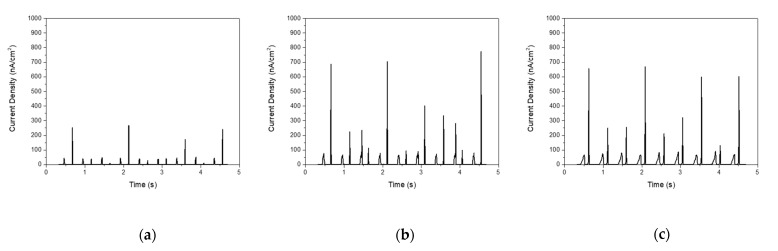
Current density generated from piezoelectric NGs with PDMS layers of various thickness, (**a**) 40, (**b**) 80, (**c**) 100 μm, on ZNRs with an aspect ratio of 11:1.

Figure 9 shows the current densities generated by the piezoelectric NGs. As the thickness of the PDMS layer increased from 40 to 100 μm, the current density increased. When ZNRs are deformed by an external force, electrical charges are induced by the piezoelectric potential difference [27,28]. The density of charges was higher in the PDMS layer with 80-μm thickness, resulting in improved current density of NGs, as summarized in Table 3. The NG with the 80-μm-thick PDMS layer had the higher current density of 773.2 nA/cm^2^, the reason being that the difference in work function between the top Ag electrode and the PDMS layer with the optimum thickness of 80 μm was higher. Compared to the ZnO composite structure, this PDMS layer was expected to protect the well-ordered ZNR layer formed on the substrate.

**Figure 10:**
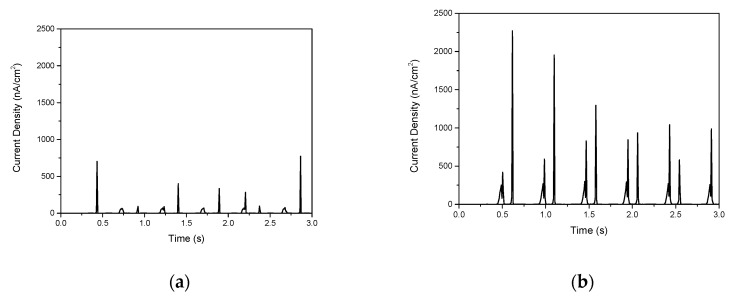
Current density of piezoelectric NGs based on ZNRs and an 80-μm-thick PDMS layer: (**a**) aspect ratio of 11:1 without PEI, (**b**) aspect ratio of 24:1 with PEI in precursor solution.

We also fabricated NGs using an 80-μm-thick PDMS layer on a ZNR layer with the two different aspect ratios of 11:1 and 24:1 using ITO/PES substrate, respectively. As shown in Figure 10a, when using ZNRs with an 11:1 aspect ratio, the current density was 773.2 nA/cm^2^. In contrast, as shown in Figure 10b, the highest current density increased to 2270.1 nA/cm^2^ for the ZNRs fabricated with a higher aspect ratio of 24:1. The ZNRs with a higher aspect ratio were easily bent under the same pressure condition, leading to the enhancement of the piezoelectric properties [29]. Although these ZNRs have a higher aspect ratio, the PDMS layer also plays an important role in protecting the ZNRs and enhancing the piezoelectric output performance in terms of higher density of charges.

## 4. Conclusions

Piezoelectric NGs were fabricated using ZNRs and PDMS layers on ITO-coated substrates. For fabrication of well-aligned ZNRs, a ZnO seed layer was successfully prepared under optimum conditions. ZNRs with various aspect ratios were fabricated on these seed layers. PEI was added into precursor solutions to obtain higher aspect ratios of the ZNRs. Consequently, ZNRs with the highest aspect ratio of 24:1 were obtained. The thickness of the PDMS layer was optimized to increase the work function difference for the top Ag electrode. It was also confirmed that the PDMS layer with an optimal thickness of 80 μm improved the charge density of the surface. As a result, piezoelectric NGs with ZNRs with the highest aspect ratio of 24:1 and 80-μm PDMS layers produced the highest current density of 2270.1 nA/cm^2^. Therefore, these piezoelectric NGs have great potential for applications such as self-powered sensors and low-power electronics.

## Figures and Tables

**Table 1:** Summarized diameter, length, and aspect ratios of ZNRs prepared with various growth times at 90 °C without and with PEI in precursor solutions.

Growth time (h)	Length (μm)	Diameter (nm)	Aspect Ratio
3	1.389	162.2	8:1
3 (w/PEI)	1.438	70.30	20:1
6	2.869	402.3	7:1
6 (w/PEI)	3.194	142.7	22:1
9	5.197	478.0	11:1
9 (w/PEI)	4.164	173.0	24:1

**Table 2:** Summarized work function of PDMS layers of various thickness, (a) 40, (b) 80, (c) 100 μm, on ITO substrate and difference in work function with Ag electrode.

Structures	Average Work Function (eV)	ΔWork Function with Ag Layer (4.64 eV)
PMDS (40 μm)/ITO/Glass	4.99	0.35
PMDS (80 μm)/ITO/Glass	5.50	0.86
PMDS (100 μm)/ITO/Glass	5.21	0.57

**Table 3:** Current density generated from piezoelectric NGs with various thicknesses of PDMS, (a) 40, (b) 80, (c) 100 μm, on ZNRs with an aspect ratio of 11:1.

Aspect Ratio	PDMS Thickness (μm)	Current Density (nA/cm^2^)
11:1	40	360.5
	80	773.2
	100	669.4

## Data Availability

The data presented in this study are available on request from the corresponding author.
